# Fingerprinting of Psychoactive Drugs in Zebrafish Anxiety-Like Behaviors

**DOI:** 10.1371/journal.pone.0103943

**Published:** 2014-07-31

**Authors:** Caio Maximino, Annanda Waneza Batista da Silva, Juliana Araújo, Monica Gomes Lima, Vanessa Miranda, Bruna Puty, Rancés Benzecry, Domingos Luiz Wanderley Picanço-Diniz, Amauri Gouveia, Karen Renata Matos Oliveira, Anderson Manoel Herculano

**Affiliations:** 1 Laboratório de Neurociências e Comportamento “Frederico Guilherme Graeff”, Departamento de Morfologia e Ciências Fisiológicas, Centro de Ciências Biológicas e da Saúde, Universidade do Estado do Pará, Marabá, Pará, Brazil; 2 Instituto de Ciências Biológicas, Universidade Federal do Pará, Belém, Pará, Brazil; 3 Laboratório de Neuroendocrinologia, Instituto de Ciências Biológicas, Universidade Federal do Pará, Belém, Pará, Brazil; 4 Universidade Federal do Oeste do Pará, Oriximiná, Pará, Brazil; 5 Laboratório de Neurociências e Comportamento, Núcleo de Pesquisa e Teoria do Comportamento, Universidade Federal do Pará, Belém, Pará, Brazil; University of Toronto Mississauga, Canada

## Abstract

A major hindrance for the development of psychiatric drugs is the prediction of how treatments can alter complex behaviors in assays which have good throughput and physiological complexity. Here we report the development of a medium-throughput screen for drugs which alter anxiety-like behavior in adult zebrafish. The observed phenotypes were clustered according to shared behavioral effects. This barcoding procedure revealed conserved functions of anxiolytic, anxiogenic and psychomotor stimulating drugs and predicted effects of poorly characterized compounds on anxiety. Moreover, anxiolytic drugs all decreased, while anxiogenic drugs increased, serotonin turnover. These results underscore the power of behavioral profiling in adult zebrafish as an approach which combines throughput and physiological complexity in the pharmacological dissection of complex behaviors.

## Introduction

Along with benzodiazepines, drugs targeting the serotonergic system represent a major class of anxiolytic drugs. Among available serotonergic drugs, selective serotonin reuptake inhibitors still represent the most prescribed treatment for anxiety disorders, even though they are associated with low efficacy in a considerable proportion of patients, a delayed onset of therapeutic action, and diverse collateral effects which reduce tolerance (e.g., sexual dysfunction, weight changes). Benzodiazepines, on the other hand, are associated with decreased responsiveness over time, withdrawal-related symptoms, and sedation [1]. The need for novel, more efficient anti-anxiety drugs is paramount [2], but the proper level for consistent results with relatively high throughput is difficult to determine [3], [4].

While in other fields of pharmaceutical discovery *in vitro* target-based assays are sufficient to accelerate discovery and increase throughput, these approaches (while certainly initially useful [5]) are unfeasible in most areas of psychopharmacology, where the appropriate targets remain unknown [4], [6]. Thus, psychopharmacological research relies on phenotype-based approaches, in which behavior is the principal endpoint [7]–[12].

A major obstacle in the discovery of psychopharmacological agents is the difficulty in predicting how candidate drugs can alter complex behaviors. While the usual approach of complete identification of the mechanisms of disease pathology is certainly useful, this strategy can preclude the discovery of novel psychoactive drugs that target unexpected processes. It has been proposed that phenotype-based approaches in the context of a whole organism are a suitable alternative to overcome these limitations [4], [7], [10]–[12], but the throughput of these assays is usually low [3], [4], [6]. Behavioral assays in mammals represent a high degree of physiological complexity in relation to *in vitro* target-based assays, but the throughput is low; conversely, *in vitro* assays are high-throughput but low-content [3], [4], [6]. Behavioral assays in larval and adult zebrafish have the potential to combine the high content of phenotype-based approaches with the medium-to-high-throughput of *in vitro* chemical screening methods [13]–[22].

Zebrafish became a widely used model organism due to its fecundity, physiological complexity, and the existence of many genetic and genomic tools [6], [16], [23]. While larval zebrafish has been proposed as an ideal model for phenotype-based behavioral assays in psychopharmacological drug discovery [24], the behavioral repertoire of developing zebrafish is considerably restricted [25], [26] and considerable neurochemical and behavioral differences exist between larvae and adults [27]–[29]. In contrast, adult zebrafish display a complete repertoire of behaviors which have been characterized physiologically and pharmacologically [17]. Among these, drug-sensitive phenotypes of anxiety, such as geotaxis [30]–[33] and scototaxis [34]–[38], have been described and pharmacologically and behaviorally validated.

Here, we describe the results of phenotyping in the scototaxis test, using adult zebrafish, as a medium-throughput, high-content assay for anxiolytic and anxiogenic drugs. Compounds analyzed included drugs with known anxiolytic effect (benzodiazepines, buspirone), drugs with known anxiogenic effect (caffeine), as well as drugs with known motor stimulating effects (diethylpropion, bupropion). In addition, drugs acting on adenosinergic (DPCPX, PACPX, ZM 241,285, and DMPX), glutamatergic (NMDA, MK-801), serotonergic (serotonin, WAY 100,635, SB 224,289, moclobemide, *Hypericum perforatum* extract) and nitrergic (L-NOARG, SNP) systems were tested. Multiple behavioral parameters were measured, including time spent in the white compartment, locomotion into and on the white compartment, and ethologically-defined endpoints such as thigmotaxis, erratic swimming, risk assessment and freezing. These endpoints were then analyzed using a clustering paradigm, used before for profiling rest/wake promoting-drugs in larval zebrafish [34], [36], [39], [40] and to analyze anxiety-like behavior and habituation in the novel tank test [41], [42].

## Results and Discussion

**Figure 1:**
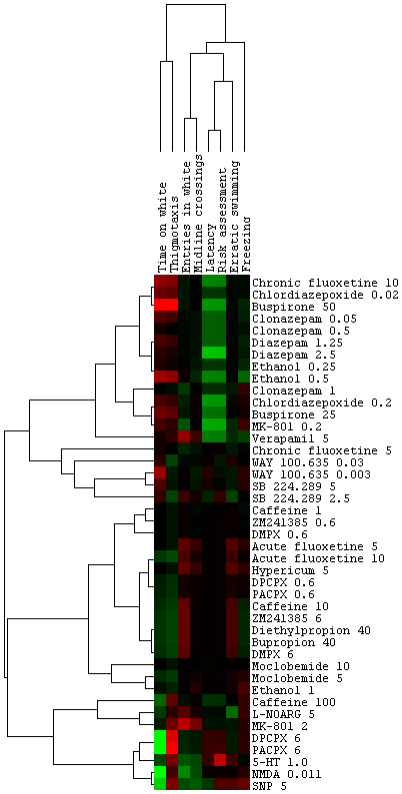
Behavioral fingerprint of selected drugs on the scototaxis test. Pharmacological manipulations were hierarchically clustered to link compounds to behaviors. In the clustergram, each cell represents the Maximum Predictive Value (red – higher than controls; green – lower than controls).

**Figure 2:**
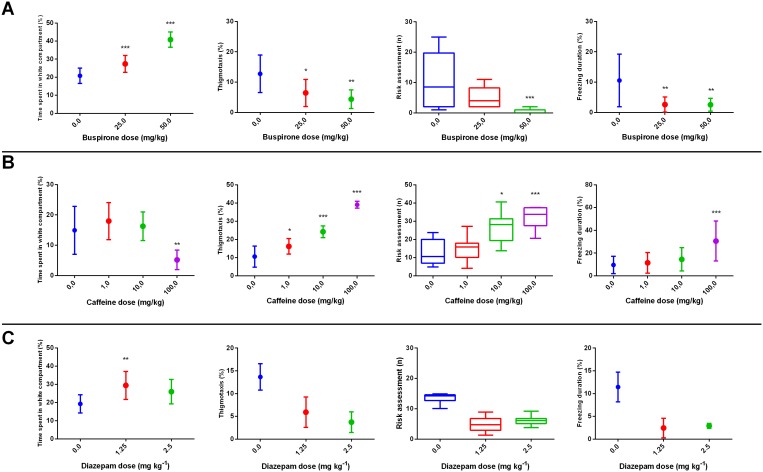
Effects of (A) buspirone, (B) diazepam and (C) caffeine on time on white (upper left), risk assessment (upper right), thigmotaxis (lower left), and freezing (lower right). Bars represent standard error of the mean, and whiskers represent the 2.5 and 97.5 percentile. *, p<0.05; **, p<0.01; ***, p<0.001.

Given the diversity of potential drug effects in the different behavioral parameters, a “behavioral fingerprint” was assigned to each compound and dose by determining the Maximum Predictive Value [15], [43] for each effect and applying clustering algorithms to organize behavioral parameters and molecules [44]. This analysis allowed the organization of the data set broadly into anxiolytic, anxiogenic, and motor stimulating, identifying four clusters which correspond to “avoidance” (Time on white and Thigmotaxis), “locomotor” (Entries in white and Midline crossings), “risk assessment” (Latency to white, Risk assessment and Erratic swimming) and “fear” (Freezing) measures (Figure 1). For example, buspirone and diazepam produced a marked anxiolytic-like effect – increasing time spent in the white compartment and decreasing risk assessment, thigmotaxis and freezing in the white compartment (Figures 2A and 2B) –, while caffeine had an opposite profile (Figure 2C).

**Figure 3:**
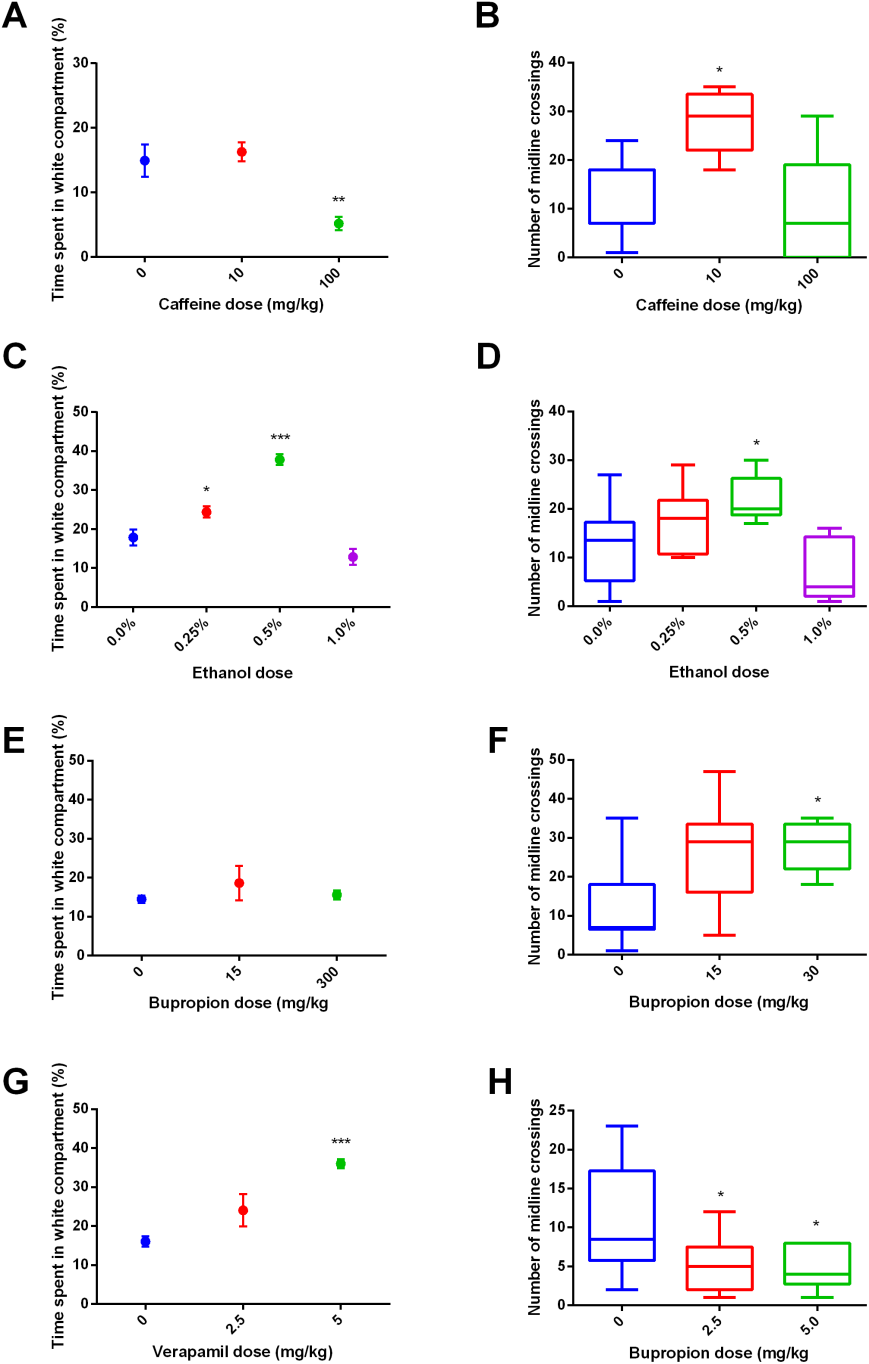
Effects of (A) caffeine, (B) ethanol, (C) bupropion and (D) verapamil on time on white (top) and entries on white (bottom). Bars represent standard error of the mean, and whiskers represent the 2.5 and 97.5 percentile. *, p<0.05; **, p<0.01; ***, p<0.001.

**Figure 4:**
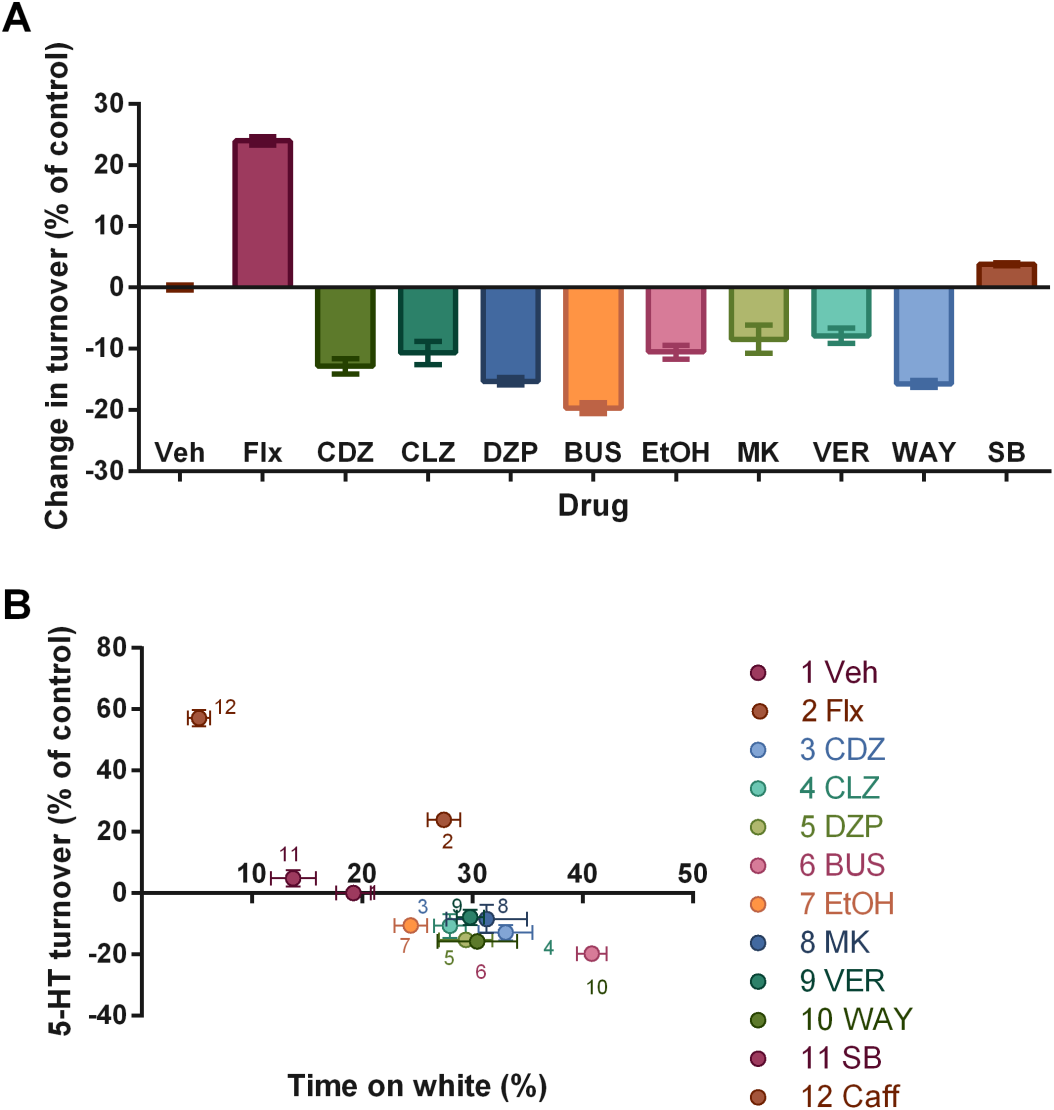
Drugs which cluster on the 'anxiolytic' group decrease 5-HT turnover in the brain. (A) Turnover rates, as measured by 5-HIAA:5-HT ratios, normalized to the values of vehicle-treated animals, for the following drugs: fluoxetine (FLX; chronic treatment with 10 mg/kg); chlordiazepoxide (CDZ; 0.02 mg/kg); clonazepam (CLZ; 0.05 mg/kg); diazepam (DZP; 1.25 mg/kg); buspirone (BUS; 50 mg/kg); ethanol (EtOH, 2.5%); dizocilpine (MK; 0.005 mg/kg); verapamil (VER; 5 mg/kg); WAY 100,635 (WAY; 0.03 mg/kg); and SB 224,289 (SB; 2.5 mg/kg). Asterisks mark statistically significant differences in relation to vehicle-treated animals (F_10, 43_ = 45.99, p<0.0001, one-way ANOVA followed by Dunnett's Multiple Comparison test). Bars represent mean (B) Correlation between turnover rates (*Y*-axis) and time spent in the white compartment (*X*-axis) for vehicle- and drug-treated animals (*n* = 4 for each point). Points represent means and error bars represent standard errors. A negative correlation is found between the decrease in serotonin turnover and the increase in time on white produced by a drug (r^2^ = 0.5688, p = 0.0073).

Cluster analysis revealed a high degree of predictive validity in the proposed assay (Figures 1–3). First, anxiolytic drugs with clinical efficacy (benzodiazepines, chronic [14 days] fluoxetine) cluster together with buspirone, and anxiogenic drugs (NMDA, 5-HTP) cluster with caffeine (Figure 1). Second, motor-stimulating drugs (which represent potential false positives in locomotor-based assays) form their own cluster (Figure 1). For example, a low dose of caffeine (Figure 3A), a low dose of ethanol (Figure 3B), and bupropion (Figure 3C) increased locomotion, without effects on ethological measures or time in the white compartment. Second, drugs with multiple targets (e.g., ethanol, caffeine) correlated with drugs in different cluster in a dose-dependent way (Figure 1); caffeine, for example, clustered with anxiogenic drugs at a higher dose, and with stimulant drugs at a lower dose (Figure 1). Third, anxiolytic/anxiogenic and locotomor stimulating effects closely followed those observed in mammals. Fourth, compounds which clustered on the “anxiolytic” effect (Figure 1) all reduced serotonin turnover, which was correlated with time spent on the white compartment in these groups (r^2^ = 0.5688, p = 0.007) (Figure 4). These analyses indicate that compounds with shared systems effects produce similar phenotypes which are conserved across vertebrates. While from a neuroanatomical and genomic point of view the serotonergic system diverges from that of mammals [45], these and other data strongly suggest that the function of the serotonergic system is conserved across vertebrates. It should also be observed that some behavioral components (time on white, thigmotaxis, latency to white and risk assessment) are more strongly affected by drug treatments (Figure 1), suggesting that those parameters have a stronger predictive value to pharmacological treatments. Interestingly, time on white and thigmotaxis cluster together, while latency to white and risk assessment fall together on another cluster. Erratic swimming and freezing, while affected by anxiogenic and anxiolytic drug treatments, show a weaker liability. These results are in accordance with those observed in the novel tank test [15], in which erratic swimming and freezing had weaker predictive power in relation to time in the upper half of the tank and latency to upper half.

Furthermore, cluster analysis revealed novel behavioral effects of poorly characterized substances. For example, the calcium channel blocker verapamil, an anti-arrhythmic and anti-anginal agent, produced a small anxiolytic effect, clustering with sedative doses of ethanol and clonazepam (Figure 1, Figure 3D). Interestingly, verapamil has been shown to be sedative in larval zebrafish [15], [41]. This effect is unlikely to be a consequence of antihypertensive effects, because sodium nitroprusside (SNP) had an opposite effect and clustered with NMDA (Figure 1).

These results reveal a conserved neuropharmacology in vertebrates and identify novel regulators of anxiety, such as the glutamatergic/nitrergic system. Previously validated targets in zebrafish anxiety assays include the cholinergic system [30], [46], [47], histamine [48]–[50], central benzodiazepine receptors [34], [51]–[54], endogenous opioids [32], [55], endocannabinoids [55]–[57], serotonin [58]–[60], and adenosine [34], [53], [61], [62]. The behavioral profiling observed in this paper is also predictive of decreased serotonin turnover, suggesting a common neurobiological mechanism of anxiolysis. This is a surprising result given that, while the effects of serotonergic drugs on zebrafish behavior seem to be rather conserved, from a genomic and neuroanatomical point of view the serotonergic system from mammals is different from that of teleosts (e.g., presence of hypothalamic and tectal serotonergic nuclei and duplicated *htr1a* and *sert* genes in zebrafish) [45]. Nonetheless, these results support a role for the serotonergic system in controlling zebrafish anxiety, suggesting conserved function, if not conserved structure.

The medium throughput of this method in relation to, e.g., larval profiling [41] is offset by the increased information content produced by analyzing multiple parameters (anxiety-like responses, dark preference, motor parameters) and using developed, adult animals. We underscore that the outstanding predictive validity of the proposed assay is also accompanied by construct validity [6], [41], [42], which enriches and directs the predictive validity of the model. Therefore, light/dark preference in adult animals can complement traditional target-based discovery methodologies, combining the physiological complexity of *in vivo* assays with medium-to-high-throughput, low-cost screening [63]. This has been done previously – albeit with a limited amount of drug treatments – with the novel tank test, with results similar to those presented here: caffeine, for example, clustered among anxiogenic manipulations, while chronic fluoxetine clustered among anxiolytic manipulations [15]. Similarly, anxiogenic treatments increase erratic swimming and freezing duration in the novel tank test [15] as well as in the present experiments. Caution should be taken, however, in generalizing results from both assays, since drug effects in the light/dark and in the novel tank tests are not always the same – and, in fact, some drugs, such as pCPA and acute fluoxetine, produce opposite effects in each test [58]. Moreover, there is substantial evidence for different stimulus control in these tests [63], reinforcing the hypothesis that they model different aspects of anxiety-like behavior. While it is not fully understood whether exposure to the light/dark test could impact latter testing with the novel tank test, in principle both tests could be used in a 'test battery' of behavioral assays. This approach could greatly increase the information content and circumvent the limitation of analyzing a small amount of variables.

In conclusion, the present work, combined with other attempts at clustering behavioral variables and treatments in adult and larval zebrafish [15], [41], [42], suggest that behavioral screening is able to characterize relatively large classes of chemical compounds, revealing differences in efficacy and side effects (e.g., sedation) that cannot be detected *in vitro*.

## Materials and Methods

### Ethical statement

Animals were housed and manipulated in ways that minimized their potential suffering, as per the recommendations of the Canadian Council on Animal Care [6]. All procedures complied with the Brazilian Society for Neuroscience and Behavior’s (SBNeC) guidelines for the care and use of animals in research, and experiments were approved by the Comitê de Ética no Uso de Animais (CEUA) from UEPA.

### Subjects and housing

430 adult zebrafish from the shortfin wild type phenotype were bought in a local ornamental fish shop and brought to the laboratory facilities, where the animals were left to acclimate for at least two weeks before experiments begun. Animals were group-housed in 40 L tanks, with a maximum density of 25 fish per tank. Tanks were filled with deionized and reconstituted water at room temperature (28°C) and a pH of 7.0–8.0. Lighting was provided by fluorescent lamps in a cycle of 14–10 hours (LD), according to the standards of zebrafish care [64].

### Drugs

Anhydrous caffeine was bought from Quimis (Diadema/SP, Brazil). PACPX, DPCPX, ZM 241385 and DMPX were bought from Research Biochemicals International (Natick/MA, USA). Clonazepam, diazepam and moclobemide were bought from Roche (Brazil). Fluoxetine hydrochloride was bought from Eli Lily (Brazil). Buspirone hydrochloride, bupropion and diethylpropion were bought from Bristol-Myers Squibb (Brazil). Ethanol was bought from Cromoline (Brazil). Chlordiazepodixe was bought from Farmasa (Brazil). NMDA and serotonin were bought from Tocris (Bristol, UK). MK-801, WAY 100635, SB 224289, sodium nitroprusside and L-NAME were bought from Sigma (Saint Louis/MO, USA). *Hypericum perforatum* hydroalcoholic extract was a kind gift from Dr. Marcelo Pereira, and prepared as follows: above-ground parts of the plant were dried for 10 days at room temperature, after which they were ground by an atomic blender; 100 g of the plant powder was soaked in 96% ethanol for 72 h and then filtered and concentrated by a vacuum distiller. The concentrated solution was decanted chloroform in three consecutive steps, and the resulting solution was vaporized and desiccated at 50°C under sterile conditions. Drugs were dissolved on Cortland’s salt solution or 1% DMSO prior to experiments, and injected intraperitoneally at a volume of 0.1 µl per mg body weight with a 10 µl microsyringe equipped with a 33G needle (Hamilton, USA). Chronic fluoxetine treatment was made by daily injections for 14 days.

### Light/dark preference

Determination of drug effects on scototaxis were carried as described elsewhere [65]. Briefly, after drug injection and effect onset animals were transferred to the central compartment of a black and white tank (15 cm×10 cm×45 cm h×d×l) for a 3-min. acclimation period, after which the doors which delimit this compartment were removed and the animal was allowed to freely explore the apparatus for 15 min. The following variables were recorded, along with the reference to their extended definition in the Zebrafish Behavior Catalog [17]:

*time on the white compartment:* the time spent in the top third of the tank (percentage of the trial) (ZBC 1.137);
*squares crossed:* the number of 10 cm^2^ squares crossed by the animal in the white compartment (ZBC 1.54);
*latency to white:* the amount of time the animal spends in the black compartment before its first entry in the white compartment (s);
*entries in white compartment:* the number of entries the animal makes in the white compartment in the whole session (ZBC 1.54);
*erratic swimming:* the number of “erratic swimming” events, defined as a zig-zag, fast, unpredictable course of swimming of short duration (ZBC 1.51);
*freezing:* the proportional duration of freezing events (in % of time in the white compartment), defined as complete cessation of movements with the exception of eye and operculae movements (ZBC 1.68).
*thigmotaxis:* the proportional duration of thigmotaxis events (in % of time in the white compartment), defined as swimming in a distance of 2 cm or less from the white compartment’s walls (ZBC 1.173).
*risk assessment:* the number of “risk assessment” events, defined as a fast (<1 s) entry in the white compartment followed by re-entry in the black compartment, or as a partial entry in the white compartment (i.e., the pectoral fin does not cross the midline).

### Maximum Predictive Value calculations

For each variable analyzed, Maximum Predictive Values (MPVs) were calculated as the ratio of the mean difference between control and treatment groups and their pooled standard deviations [40]:
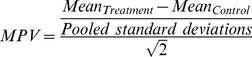
Where pooled standard deviations are defined as
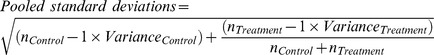



### Clustering algorithm

After calculation of MPVs, these values were input into Cluster 3.0 (University of Tokyo, Japan), where hierarchical clustering was performed across behavioral endpoints and drug treatments and doses. Data were centered around the median, and clustering was then made using Spearman Rank Correlation with Average linkage as similarity metric. Clustering results were then visualized as dendograms and colored arrays in Java TreeView (University of Glasgow, UK).

### HPLC analysis of indoleamines

Serotonin and 5-HIAA (5 mg) were dissolved in 100 mL of eluting solution (50 ml MilliQ water, 0.43 ml HClO_4_ 70% [0.2 N], 10 mg EDTA, 9.5 mg sodium metabissulfite) and frozen at −20°C, to later be used as a standard.

The HPLC system consisted of a delivery pump (LC20-AT, Shimadzu), a 20 µL sample injector (Rheodyne), a degasser (DGA-20A5), and an analytical column (Shimadzu Shim-Pack VP-ODS, 250×4.6 mm internal diameter). The integrating recorder was a Shimadzu CBM-20A (Shimadzu, Kyoto, Japan). An electrochemical detector (Model L-ECD-6A) with glassy carbon was be used at a voltage setting of +0.83 V, with a sensitivity set at 8 nA full deflection. The mobile phase consisted of a solution of 70 mM phosphate buffer (pH 2.9), 0.2 mM EDTA, 5% methanol and 20% sodium metabissulfite as a conservative. The column temperature was set at 17°C, and the isocratic flow rate was 1.8 ml/min. 0.5 mL of extracellular fluid (ECF) were extracted by quickly removing one brain from the skull and incubating it in 2 mL of 50 mM TBS, pH 7.4, containing 90 mM NaCl, 2.5 mM CaCl2, 1 mM glutathione for 30 min at 4°C (*7*). This fluid was then mixed with 0.5 mL of eluting solution, filtered through a 0.22 µm syringe filter, and then injected into the HPLC system.
